# The Immature Gut Barrier and Its Importance in Establishing Immunity in Newborn Mammals

**DOI:** 10.3389/fimmu.2020.01153

**Published:** 2020-06-09

**Authors:** Björn Weström, Ester Arévalo Sureda, Kateryna Pierzynowska, Stefan G. Pierzynowski, Francisco-José Pérez-Cano

**Affiliations:** ^1^Department of Biology, Lund University, Lund, Sweden; ^2^Precision Livestock and Nutrition Unit, TERRA Teaching and Research Centre, Gembloux Agro-Biotech, University of Liège, Gembloux, Belgium; ^3^Department of Animal Physiology, Kielanowski Institute of Animal Physiology and Nutrition, Jablonna, Poland; ^4^Department of Medical Biology, Institute of Rural Health, Lublin, Poland; ^5^Physiology Section, Department of Biochemistry and Physiology, Faculty of Pharmacy and Food Science, University of Barcelona, Barcelona, Spain; ^6^Research Institute of Nutrition and Food Safety of the University of Barcelona (INSA-UB), Santa Coloma de Gramenet, Spain

**Keywords:** intestine, permeability, fetal, transcytosis, FcRn, tolerance, immunity

## Abstract

The gut is an efficient barrier which protects against the passage of pathogenic microorganisms and potential harmful macromolecules into the body, in addition to its primary function of nutrient digestion and absorption. Contrary to the restricted macromolecular passage in adulthood, enhanced transfer takes place across the intestines during early life, due to the high endocytic capacity of the immature intestinal epithelial cells during the fetal and/or neonatal periods. The timing and extent of this enhanced endocytic capacity is dependent on animal species, with a prominent non-selective intestinal macromolecular transfer in newborn ungulates, e.g., pigs, during the first few days of life, and a selective transfer of mainly immunoglobulin G (IgG), mediated by the FcRn receptor, in suckling rodents, e.g., rats and mice. In primates, maternal IgG is transferred during fetal life via the placenta, and intestinal macromolecular transfer is largely restricted in human neonates. The period of intestinal macromolecular transmission provides passive immune protection through the transfer of IgG antibodies from an immune competent mother; and may even have extra-immune beneficial effects on organ maturation in the offspring. Moreover, intestinal transfer during the fetal/neonatal periods results in increased exposure to microbial and food antigens which are then presented to the underlying immune system, which is both naïve and immature. This likely stimulates the maturation of the immune system and shifts the response toward tolerance induction instead of activation or inflammation, as usually seen in adulthood. Ingestion of mother's milk and the dietary transition to complex food at weaning, as well as the transient changes in the gut microbiota during the neonatal period, are also involved in the resulting immune response. Any disturbances in timing and/or balance of these parallel processes, i.e., intestinal epithelial maturation, luminal microbial colonization and mucosal immune maturation due to, e.g., preterm birth, infection, antibiotic use or nutrient changes during the neonatal period, might affect the establishment of the immune system in the infant. This review will focus on how differing developmental processes in the intestinal epithelium affect the macromolecular passage in different species and the possible impact of such passage on the establishment of immunity during the critical perinatal period in young mammals.

## Introduction

The gut, in addition to its primary function of nutrient digestion and absorption, constitutes an effective barrier to protect against the invasion of pathogenic microorganisms and passage of potential harmful macromolecules into the body. However, as opposed to the restricted macromolecular passage in the adult, enhanced transfer of macromolecules across the immature intestinal epithelium takes place during the fetal and neonatal periods (1). The high intestinal permeability during these periods is due to the high endocytic capacity of the immature (fetal-type) enterocytes (2–4). These fetal-type enterocytes internalize luminal content containing macromolecules, by fluid-phase or receptor-mediated endocytosis, either for intracellular digestion in digestive vacuoles or for their vesicular transfer through the cell and release on the basolateral side (transcytosis). The intestinal transfer can either be non-selective, with uptake and passage of an array of luminal macromolecules, or the transfer can be more selective due to epithelial expression of the neonatal Fc (fragment crystallizable) receptor (FcRn) that binds and mediates the transepithelial transfer of immunoglobulin G (IgG) (5–11).

This property of the intestinal epithelium is largely lost with the progress of development and maturation, during the fetal and neonatal periods, until macromolecular transepithelial transfer ceases at the time of the so-called “gut closure.” In the mature intestine, after gut closure, some macromolecular transfer still takes place, but the extent of transfer is limited and it is restricted in terms of intestinal area, with transfer occurring mainly in the follicle-associated epithelium, especially over the membranous cells (M-cells), overlaying the immune-cell rich regions in the small intestine known as the Peyer's patches (12). Recent research also suggests passage of antigenic molecules through intestinal goblet cells (13). In addition, a paracellular leakage of larger molecules after an inflammatory-induced opening of the epithelial tight junctions may also occur (14).

This review will focus on the developmental processes taking place in the intestinal epithelium, highlighting the high macromolecular passage during the critical perinatal period and the impact of such passage on the development of immunity in the young, with possible effects later in life. In addition, the review will describe the differences in timing and amount of such macromolecular transfer between species, in particular those used as animal models for humans, and the impact this could have when comparing different species.

## Enhanced Intestinal Macromolecular Transfer in Fetal/Neonatal Life

The extent and time period of macromolecular transfer over the immature intestine, before gut closure, is dependent on animal species (1). In eutherian (placental) species, this appears to be linked to the type of placentation and number of tissue layers separating the fetal and maternal blood circulations, hence affecting the extent of the macromolecular transfer between mother and offspring. In non-mammalian vertebrates, such as fish and birds, maternal macromolecular transfer takes place from the yolk, over the yolk sac endoderm, and finally to the offspring's blood circulation (1, 15, 16). In mammalian species, such as rodents, the yolk sac is everted and participates in some macromolecular transfer from the uterus, however, the immature neonatal gut takes over this role and constitutes the main route of macromolecular transmission in rats and mice (1, 4). In other mammalian species, such as ungulates and primates, the yolk sac either atrophies during gestation or is absent (17). In ungulates macromolecular transfer occurs from the first milk, known as colostrum, via the immature gut for a short period after birth, while in primates the main macromolecular transfer is occurs via the placenta and intestinal macromolecular transfer is largely restricted to fetal life (Table 1).

**Table 1:** Overview and comparison of macromolecular transmission between mother and offspring in different species.

**Taxon/species**	**Pre- or Postnatal**	**Gestation length**	**Maternal source**	**Route of transfer to offspring**	**Transfer mechanism**	**Gut closure age**	**Weaning age**	**Age at immune maturity**	**Literature**
Aves (chicken)			Yolk	Yolk sac endoderm	Receptor-mediated endocytosis, FcRY/IgY	–	–	>2–3 weeks	(1, 16)
Lagomorphs (rabbit)	Prenatal	3–4 weeks	Uterine secretion	Everted yolk sac	Receptor-mediated endocytosis, FcRn/IgG	–	5–6 weeks	After Infancy (>6 weeks)	(18–21)
Rodents (rat, mouse)	Prenatal	3 weeks	Blood	Everted yolk sac	Receptor-mediated endocytosis, FcRn/IgG	–		After Infancy (>6 weeks)	(9, 20, 22–28)
	Postnatal		Milk	Proximal small intestine	Receptor-mediated endocytosis, FcRn/IgG	3 weeks	2–3 weeks		
Rodents (guinea pig)	Prenatal	8–10 weeks	Uterine secretion	Everted yolk sac	Receptor-mediated endocytosis, FcRn/IgG	–	4–6 weeks	Post weaning (>6 weeks)	(15, 20, 29–31)
Ungulates (pig, sheep)	Prenatal	16 weeks						Post weaning (>7 weeks)	(32–36)
	Postnatal		Colostrum	Proximal small intestine	Macropinocytosis, (FcRn)	1–2 days	4–12 weeks		
Carnivores (cat, dog)	Prenatal	9 weeks (dog)	Blood	Placenta				After infancy (>6 months)	(37–39)
	Postnatal		Milk	Small intestine	Macropinocytosis	1–2 days	4 weeks		
Primates (human)	Prenatal	9 months	Amniotic fluid	Small intestine	Macropinocytosis, FcRn	≈22 weeks	4–6 months	After infancy (>10 years)	(4, 5, 7, 8, 40–46)
			Blood	Placenta	Receptor-mediated endocytosis, FcRn/IgG	–			

*Summary data including, pre- and/or postnatal transfer period, gestation length, maternal source, transfer route and mechanism of transfer, i.e., selective transfer of antibodies (IgG or IgY) by receptor-mediated (FcRn or FcRY) endocytosis or by macro-pinocytosis (free-fluid endocytosis). The age at gut closure, i.e., cessation of transepithelial macromolecular passage, age at weaning from mothers milk to a complex diet and approximative age of reaching immune maturity is also indicated*.

### Lagomorph and Rodent Species

In the lagomorph rabbit and in precocious rodent species, i.e., the guinea pig, macromolecular transfer takes place from uterine secretion via the everted yolk sac during the fetal period (15, 29). Even though the yolk sac endoderm may endocytose macromolecules indiscriminately, this transfer is selective since the yolk sac endoderm expresses FcRn that binds and mediates the transfer of IgG to the fetal circulation (18, 19). Thus, guinea pigs are born relatively mature, equipped with their mother's IgG repertoire, and intestinal macromolecular transfer is negligible postnatally (20, 22).

In altricial rodents such as rats and mice, similar to guinea pigs, some macromolecular transfer takes place *in utero* during the late fetal period, via the endocytic cells of the everted yolk sac endoderm. However, this is quantitatively less important compared to the postnatal intestinal transfer (9, 23), which is selective and occurs during the entire suckling period until weaning (24, 25).

The macromolecular uptake and transepithelial transfer takes place with regional differences along the small intestine. In the proximal part (jejunum), highly endocytic fetal-type enterocytes express FcRn receptors that bind and mediate the transepithelial transfer of IgG, as well as minor quantities of other milk proteins (24, 25, 47–49). The intestinal expression of the FcRn receptor is consistent with the high percentage of IgG (~80% of total Ig) present in rodent milk in comparison with that of human breast milk (~10%) (50). In fact, intestinal FcRn expression in premature rat pups is higher than that observed in term rats, suggesting a compensatory mechanism to counteract the low IgG passage during the fetal period (51).

In contrast, in the distal small intestine (ileum), the fetal-type epithelium internalizes luminal material via the apical endocytic complex and forms large digestive vacuoles that make up most of the cytoplasmic content (26, 52), allowing little macromolecules to pass the epithelium intact. The endocytosis machinery in mouse ileal enterocytes was recently identified and described as consisting of the multi-ligand scavenger (protein) receptors, Cubulin and Amnionless, together with the adaptor protein, Dab2, as mediators of the endocytosis mechanism (53, 54). In fact, these highly endocytic intestinal cells equipped with this multi-ligand endocytic machinery were also found in the zebrafish, indicating a conserved presence and function in vertebrates. Thus, instead of mediating transepithelial transfer of macromolecules; the cells of the rodent distal small intestine play a nutritional role with intracellular digestion, especially of protein, sustaining the rapid post-natal growth.

At about 2 weeks of age, when pups open their eyes and start to become interested in nibbling solid food in addition to suckling milk, adult-type epithelial enterocytes with drastically reduced endocytic capacity and decreased FcRn expression appear in the crypts and move up the villi, in both the proximal and distal parts of the small intestine. By 3 weeks of age, at weaning (27), this maturation process has finished and gut-closure is completed, so all fetal-type enterocytes have been replaced by the adult-type epithelium (49, 55–58).

Precocious intestinal maturation may be induced by premature weaning (59) or by luminal stimulation of suckling rats by, e.g., exposure to the lectin, phytohaemagglutinin (PHA), binding to the mucosa (60); experimental feeding of the polyamine, spermine (61, 62); administration of exogenous corticosteroids (63, 64) and by provocation with proteases (65). All these treatments stimulate crypt-cell proliferation, and thus, increase intestinal epithelial cell turnover and renewal to adult-type enterocytes with heavily decreased endocytic activity and macromolecular transfer capacity along the villi.

### Ungulate (Hoofed) Species

In ungulate species, the epitheliochorial placenta consists of four epithelial/endothelial layers between the maternal and the fetal circulations, which constitutes an impermeable and effective barrier to macromolecules. Therefore, ungulates, i.e., piglets, lambs, calves and foals; are agammaglobulinemic at birth and during the first 1–2 days of life they display an extensive macromolecular transmission, including that of colostral antibodies over the intestines, making suckling essential for survival (32). The macromolecular transfer takes place by free-fluid endocytosis (macro-pinocytosis) in the proximal part of the small intestine and it is essentially a non-selective process. The enterocytes in the distal small intestine are also highly endocytic but the vesicles coalesce and form giant cell vacuoles designated for intra-cellular digestion and little, if any, of the macromolecules will survive intact and reach the basolateral side (4, 33). The period of intestinal transmission in newborn ungulates (piglets) matches the maternal (sow) production of colostrum, which is not only rich in nutrients, but also has a high content of IgG and other bioactive proteins. The FcRn receptor has been identified in the intestinal epithelium in piglets and lambs (34, 66) but it does not seem to be essential for the transfer of IgG, since a variety of macromolecules, including non-proteins like polyvinyl pyrrolidone (Mw 60.000) (67) and FITC-dextrans (Mw 3.000–70.000) (35, 68), can be absorbed. During this period, transfer of intact IgG and other colostrum whey proteins is facilitated by the high colostral protein content, that *per se* stimulates endocytosis (35), as well as the presence of a specific colostrum protease inhibitor (SCTI), which in turn decreases luminal protein degradation (69, 70).

At 1–2 days of life, if feeding has been initiated, gut closure takes place and the epithelium in the proximal small intestine loses its macromolecular transfer capacity (1, 68). The exact mechanism is not yet known, but the luminal proteolytic activity increases as the colostrum inhibitor level rapidly decreases, and the intestinal epithelial cells either lose their highly endocytic capacity or this capacity is limited and ceases after being fully utilized. In contrast, the endocytic capacity of the epithelium in the distal small intestine remains until up to 3 weeks of life and endocytosis of luminal material for intracellular vacuolar digestion continues (4, 33) until the epithelium of the entire small intestine is exchanged to adult-type epithelium and the endocytic capacity ceases. Thus, in ungulate species, macromolecular closure occurs early after birth in the proximal intestine without substantial cell replacement and unrelated to the weaning process.

### Carnivore Species

In cats and dogs some maternal IgG is transmitted over the placental structures to the fetus, but the main macromolecular transfer occurs postnatally from colostrum, in a similar manner to that of ungulate species (37, 38). Characteristically, the intestinal epithelium is heavily vacuolated, indicating extensive endocytosis and closure of transepithelial macromolecular transfer occurs 1–2 days after birth. After this, macromolecular uptake and intracellular vacuolar digestion continues for some weeks until these epithelial cells are replaced and intestinal maturation is completed.

### Primate Species

In contrast to the previously discussed mammalian phyla, primates, i.e., humans, show profound transfer of immunoglobulins already during the fetal period, across the hemochorial (chorioallantoic) placenta. Due to the “simple” human placenta being formed by one epithelial layer between fetal and maternal circulations, it allows for maternal-fetal macromolecular transfer. This transfer is selective and takes place by receptor-mediated transcytosis, binding of IgG to FcRn, across the syncytiotrophoblast cells (71). The transmission of IgG is low during the early fetal period (1st and 2nd trimesters) but increases significantly during the late fetal period (3rd trimester). Hence, premature infants are born with lower IgG levels in the blood than full term infants.

In addition to the major placental IgG transfer, some macromolecular passage can also take place *in utero* over the intestine, since vacuolated enterocytes have been observed in the mid-distal intestine from about 13–14 weeks of fetal life (72) and a capacity for endocytosis has been described (40). Moreover, FcRn receptors have been identified in the apical membrane of intestinal epithelial cells and are able to bind IgG molecules present in the amniotic fluid after swallowing, mediating their endocytic transfer to the fetus (5, 8, 41). This capacity is lost at mid-gestation since the fetal-type enterocytes are exchanged for enterocytes lacking endocytic properties and gut closure appears to occur already *in utero* in humans (4, 40). At birth, full term neonates are equipped with an essentially adult-type intestinal epithelium, with low expression of the FcRn receptor and thus the endocytic capacity is largely lost. Hence, macromolecular transfer in the newborn is low, albeit somewhat higher than in the adult (42). The oral sugar (lactulose/mannitol) test has indicated increased intestinal permeability for a short period of about 1 week after birth, which can be prolonged by prematurity or formula-feeding (43, 73–76). Since the intestinal epithelium still expresses some FcRn receptors, it has been suggested that luminal IgG/antigen complexes might be transported over the intestinal barrier into the lamina propria, to interact with antigen presenting cells and other immune cells (44, 77).

In general, different strategies for macromolecular transfer from mother to offspring have evolved, from taking place via the yolk sac endoderm and/or fetal intestine to occurring after birth via the immature intestine, and lastly mainly via the placenta in primates (4). Nonetheless, all mammalian species undergo a period of high endocytic activity of the immature intestinal epithelium and/or yolk sac epithelium, which are all cells of endodermal origin, allowing for an enhanced transcellular transfer of maternal macromolecules to her progeny. Mostly, it is a selective transfer of IgG, shuttled and regulated by epithelial expression of FcRn, to secure the transfer of protective maternal immunity. However, dietary and microbial macromolecules can also be transmitted, either as part of a complex with IgG, selected due to the maternal antibody specificity, or in free antigenic form by non-selective endocytosis. In all cases this period of decreased epithelial barrier function with increased transfer of maternal antibodies and environmental antigens will have an impact on the activation and maturation of the submucosal immune system.

## The Immature Immune System in Fetal/Neonatal Life

The formation of the immune system in humans starts in the embryo, develops during fetal life and only reaches maturity some years after birth (78). However, the precise organization and functionality of the early life, immature immune system is still poorly understood, both at systemic and local levels.

### A Species Comparison

The formation and development of the immune system in rodents is delayed compared to humans, due to their short gestation period (3 vs. 42 weeks, respectively) and consequently, their relative immaturity after birth. Thus, it is established that the lymphoid architecture forms prenatally in humans while it occurs mainly during the postnatal period in rodents, and for that reason newborn rodents resemble and can be used as a model for prematurity in humans. However, overall, in both cases, they develop with similar schemes (79).

In rodents, and particularly in rats, development of inductor lymphoid aggregates, as Peyer's patches, and effector immune cells found scattered in the epithelial layer (i.e., Intraepithelial lymphocytes, IEL) or in the lamina propria (i.e., Lamina propria lymphocytes, LPL), have been well characterized (28). Almost all major IEL and LPL subsets identified in adults are already present in suckling rats, but in different proportions. After birth, both IELs and LPLs expand in numbers based on the generation of precursors and their migration from the thymus to peripheral tissues, especially increasing during the second week of life which coincides with the contact with new antigens due to the start of solid food ingestion or weaning, which has been proven crucial (80, 81). Intestinal IgA production is very low during early life and the number of IgA-secreting B-cells at the end of the suckling period is far lower than that in adult rats (81). Many of these immune features are even less developed when rats are born prematurely (51, 82).

It has been proposed that T-cells are mediators of intestinal epithelial cell differentiation and they contribute to the maintenance of the epithelial barrier function (83). It is also known that T-cells, in the neonatal intestine of mice, are inhibited by T-regs and IgA-mediated antigen translocation (84). Innate lymphocytes (ILCs) in the GI tract have been suggested to constitute the counterpart of T-lymphocytes in innate immunity (85), with a role as integrative factors being both receivers and regulators of multisystem signaling in the gut (86). Noteworthy, in the neonatal intestine, a specific type of ILCs (type 3) has been described as the first immune cell colonizer (87), and they have been proposed to function as inhibitors of the immune response in early life (88).

In ungulate pigs, the complex maternal-fetal placental separation prevents the transfer of maternal immune components, such as Ig or cytokines, to the fetus, thus making intestinal passage during suckling essential for development of the immune system. The structure of innate immunity in pigs is similar to that of other mammals, with natural killer cells (NK) being the key cell-type in early life, even though their functionality is only achieved after birth when immunoregulatory bioactive molecules appear during breastfeeding (36). Although there is significant lymphopoietic activity in the fetal liver, bone marrow and thymus, the adaptive immunity at birth is comprised mostly of immature, low effector/memory T cells and mainly un-primed B–cells. Accordingly, the mucosal adaptive immune system in the neonatal piglet is also immature (36).

In human fetuses, B- and T-cells are found in the intestine as early as between 12 and 14 weeks gestation and their abundance and maturation increases until birth (89). It has been recently demonstrated that both the innate and adaptive immune systems are present as early as 16 weeks of gestation. Furthermore, fetal leukocytes display a distinctive differential clustering compared to neonatal intestinal tissues and have established T-cell and B-cell receptor diversity. In addition, most effector memory cells have a tissue resident memory (TRM) phenotype and secrete TNF-α and IFN-γ (90). Despite this, the low levels of immune cells observed in the intestine during early life, among them TRM cells and NK cells, with a lower proliferative or cytotoxic response of T cells, and dysregulated cytokine or lower IgA production, indicates a general immaturity of the immune system, leading to increased susceptibility to infections (91).

In early life, the immaturity of the immune system is paralleled with the immaturity of the intestinal barrier function, resulting in a higher passage of antigens across the intestine. Thus, within this particular time period it is essential that a balance is reached between avoiding infection while at the same time activating the immune system and inducing antigen tolerance.

### Immunomodulatory Breast Milk Components

In all mammalian neonates, breast milk is the principal nutrient source, but also the main driving factor in the maturation, activation and expansion of immune cells, particularly at mucosal sites. Even though 87% of breast milk content is water, the remaining 13% contains nutrients, as well as important bioactive compounds that have beneficial, non-nutritional functions (92). These compounds include, in addition to IgG in some species as discussed above, secretory IgA, antimicrobial factors like lactoferrin and lysozyme, cytokines, growth hormones and digestive enzymes, among others (43, 93–95). These milk-borne bioactive compounds are involved in the acquisition and appropriate establishment of the newborn's intestinal microbiota and immune system development (96). Secretory IgA (sIgA) in particular, contributes to shaping the microbiota during the suckling period and the breast milk oligosaccharides modulate the infant's microbiota by promoting the selective growth of health beneficial bacteria, like bifidobacteria, in the intestine.

Maternal immune cells are also present in breast milk and have been shown to be able to survive and pass the intestinal epithelial barrier and translocate into the newborn's tissues (97–101). Transfer of maternal immune cells may also occur during gestation, when maternal and fetal cells are exchanged across the placenta (102). However, this fetal cell transfer is quantitatively less marked than that which occurs following the ingestion of colostrum, due to its high content of immune cells. Furthermore, translocation of immunocompetent cells from mother to offspring has been shown to provide offspring with effective immunity and has been shown to have modulatory effects on the immune response in the young, thus influencing the development of their own immune system (103).

Breast milk contains an array of cytokines and growth factors involved in the newborn's immune system development, i.e., IL-6 stimulates differentiation of IgA-producing cells; IL-10 promotes tolerance to dietary and microbiota derived antigens, downregulates inflammation and promotes healing of damaged intestinal cells (104). In addition, the presence of pro-inflammatory cytokines, such as IL-8 and TNF-α, has also been reported in human milk. The growth factors in breast milk, TGF-β1 and TGF-β2, promote functional development of the gastrointestinal mucosa and are immunoregulators, whereas the epidermal growth factor (EGF) participates in tissue repair and growth and may contribute to the tightening of intercellular junctions (102). The cytokine profile of breast milk changes during the lactation period with differences in relative cytokine content in colostrum, transition milk and mature milk and, in addition, breast milk composition is also adapted to the delivery period, being different in term, preterm and very preterm neonates (104). Some of these bioactive factors, such as the EGF, have a low intestinal absorption ratio and they function mainly in the development of the gut and mucosal immune system, while others such as IL-2, are mostly absorbed and by entering the systemic circulation they can influence the maturing immune system (105).

Recently, the microbiomes of breast milk from humans (106), rats (50) and pigs (107) have been described. Milk microbiota have been suggested to function in the establishment of the infant's gut microbiota through the initial inoculum of the GI tract with microorganisms, and thus has been named “mother nature's prototypical probiotic food.” It is estimated that infants consuming about 800 mL of breast milk per day obtain ~10^5^-10^7^ bacteria from the milk (108, 109). Thus, the maternal microbiome and maternal diet influences not only the composition of the breast milk, but also the composition of the infant's microbiome (110, 111).

### Effects of Microbial Colonization

The colonization and expansion of beneficial bacteria in mucosal tissues during early life is not only important in the reduction of enteric infections, through competition with pathogens for the ecological niche and the production of antimicrobial agents or improvements in intestinal barrier function (108), but also through the stimulation and education of the host's immune system (109).

Among the many factors involved in the maturation of the intestinal immune system during early life, the colonization and establishment of a dynamic and appropriate microbiota is crucial. Exposure to microbes during early childhood is associated with protection from immune-mediated diseases later in life by permanently impacting the function of natural killer T (NKT) cells (112). NKT cells are a subset of cells which connect the innate and acquired immunity essential in early life (113) and are related to the development of allergic diseases in human subjects and mice (114). Additionally, similar levels of receptors that recognizes microbes and microbial components, such as the toll-like receptors (TLRs), have been found in human neonates and adults, although their activation causes immune reactions that are not as strong during early life (115). In fact, dysbiosis in infants seems to correlate with altered immune shaping and a chronic pro-inflammatory state later in adulthood (116).

In newborns, microbial antigens from the intestinal environment cannot be counteracted by a typical antibacterial and antiviral T helper type 1 (Th1) cell response, they induce a T helper type 2 (Th2) response instead, activating T cells and neutrophils via interleukin 8 (IL-8) (117). In contrast, an infant's dysbiotic microbiota would promote the Th1 response involving the production of pro-inflammatory cytokines, such as IL-12 and interferon (IFN)-γ (110). Regulation of the immune response seems to involve myeloid-derived suppressor cells that are already present in early life, which have demonstrated a strong ability to suppress T cells in mice and humans (118). These specific actions of the intestinal immune system limits continuous inflammatory damage and allows for intestinal colonization by the microbiota.

Overall, the time window for appropriate host-microbe cross-talk and therefore immune imprinting by microbiota, mainly takes place from birth to weaning, a period that involves a large expansion and diversification of the intestinal microbiota (119) and which coincides with the time window of increased antigen passage across the intestine.

The microbial and immunomodulatory components, at both the maternal and infant levels, are dependent on several factors which are considered as positive or beneficial when they promote the growth of bacterial genera, such as lactobacilli and bifidobacteria. Factors which positively shape the neonatal microbiome include vaginal delivery, maternal health status, long duration of lactation and the absence of antibiotic treatment during both pre- and post-natal periods. In contrast, factors that cause alterations in the infant's microbiota are C-section delivery, maternal disease status, especially infections that require antibiotics use; or absence of lactation that results in formula feeding (106, 110, 120, 121). These negative factors, such as antibiotics, not only reduce the previously mentioned bifidobacteria and lactobacilli beneficial bacterial groups, but also seem to affect other bacterial groups such as Proteobacteria, Firmicutes or the Clostridium cluster XIVa (121).

The microbial colonization of the intestine after birth will generate a diversity of new antigens that will play an important role in stimulating epithelial function and establishing the offspring's immune system (122). Weaning, with the loss of milk-born sIgA and other anti-microbial factors and the transition from mothers' milk to a complex diet will have a major impact on the dynamic microbiota development and thus in the resulting immune response during the neonatal period.

## Impact of Intestinal Macromolecular Transfer in the Fetal/Neonatal Period

**Figure 1:**
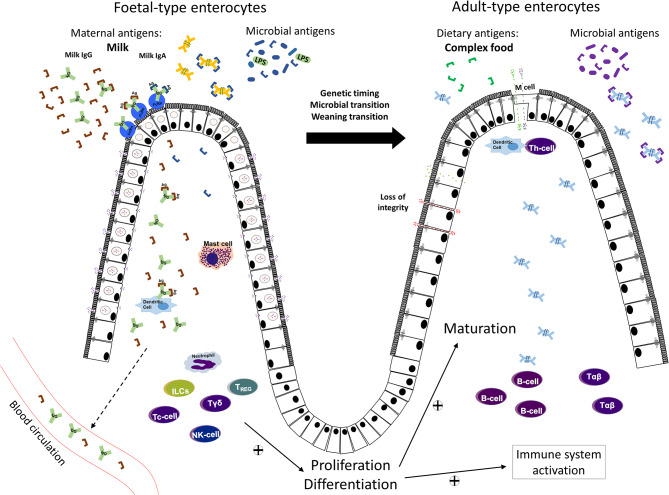
Schematic illustration of the maturational changes taking place in the small intestine, with the replacement of endocytic fetal-type epithelial cells by mature adult-type cells lacking these features during fetal/neonatal development of young mammals. Maternal macromolecules/antigens in amniotic fluid or milk (colostrum), as well as microbial antigens, may pass over the fetal and neonatal epithelial barrier and activate the underlying immature immune system. The transfer of maternal IgG is facilitated by receptor-mediated endocytosis by epithelial FcRn receptors. With maturation, at gut closure, epithelial cell endocytosis ceases and the transepithelial macromolecular passage is largely restricted. In addition to low physiological antigen passage over the adult-type epithelium, a loss of barrier integrity due to e.g., inflammation, can increase the passage of dietary or microbial antigens and impact the immune system.

At birth, when the offspring leaves the safety of the mother's womb, the intestinal barrier function is of utmost importance due to its dual function in preventing the passage of potentially harmful molecules and selectively allowing bioactive molecules and antigens to pass through. The barrier function is accomplished by several components including the luminal content, formed by endogenous secretions; the microbiota and the mucus layer; the intestinal epithelium itself, and the underlying immune system. The intestinal epithelium, a single layer of polarized cells, forms a key portion of the selective barrier, due to the endocytic capacity of the immature intestinal epithelium to select and shuttle those molecules which are allowed to pass through and reach the sub-mucosa and general circulation. Among the important macromolecules that require selective passage are the maternal antibodies, especially IgG, as well as the bioactive components in the amniotic fluid and breast milk and the microbial and dietary antigens (Figure 1).

### Passive Immune Protection by Maternal Antibodies (IgG)

The transfer of antibodies from immune competent mothers, either during fetal life or after birth, independent of route, provides newborns with effective immune protection, especially taking into account that they will most likely be exposed to the same environmental pathogens. These passively acquired maternal antibodies enter the blood circulation and provide the same protective function as actively produced antibodies. The benefit of acquiring the mother's cumulative antibody repertoire has been shown in both human and animal studies (1, 123, 124). For example, newborn husbandry ungulates, e.g., lambs or piglets, that for some reason are not able to or are not allowed to suckle and are thus deprived of naturally receiving the IgG in colostrum, will rapidly weaken and will usually develop post-natal diarrhea and succumb (125). In human neonates, since IgG is already transferred across the placenta, difficulties with regards to breast-feeding has less dramatic consequences. Even children that are genetically incapable of producing IgG (X-linked agammaglobulinemia) are protected against most infections for a few months by maternal antibodies. However, antibodies, like all proteins, are metabolized over time and IgG declines to non-protective levels, rendering these children extremely sensitive to infections (126). Previous studies in both rodents and humans have shown that maternally acquired IgG can also be transported back into the intestinal lumen, from the circulation, via the FcRn receptor (44). This can exert local protective effects, which are especially important in humans, in instances where milk contains low levels of IgG or when infants are fed with formula (127).

In addition to immune protection, passively acquired maternal antibodies reduce the metabolic costs of generating an active immune response in the offspring. Thus, passive immunity minimizes growth-suppressive effects that may be caused by activation of the immune system in species with rapid growth after birth (128). However, there is also a drawback with regards to the transfer of maternal antibodies, since it can inhibit active immunization and cause problems concerning the efficacy of vaccination of young infants, as described in both in human and veterinary medicine (129).

Recent results also indicate the existence of extra-immunological effects of maternal antibody transfer in addition to the classical protective immune effects, including direct effects on growth and functional development of the brain, the gut and possibly other organ systems in the neonate (130–132). The additional properties of circulating maternal Ig could have an impact on human neonates, especially those born prematurely with low plasma IgG levels.

### Effects of Transfer of Antigenic Macromolecules

In addition to the passive immunization by maternal transfer of antibodies (IgG), milk cells and bioactive molecules; enhanced macromolecular passage over the intestines during the fetal and neonatal periods also results, although with some variation in quantity and timing between various species, in increased microbial and dietary antigen exposure and presentation to the underlying immature immune system. The outcome of antigenic exposure and the resulting immune response during this critical time window will be dependent on the maturity of the immune system and can either result in effector cell activation or tolerance induction.

#### Gut Luminal Effects on Antigen Transfer

During the fetal and neonatal periods, intestinal luminal digestion is generally low due to the immature stomach and pancreatic function and the presence of milk-borne protease inhibitors (27, 55, 133). The reduced luminal enzymatic activity during these periods functions to keep molecules intact until they reach the intestinal epithelium, thus preserving antigenicity and possibly increasing the extent of their subsequent transepithelial passage (134). Antenatally, components in the amniotic fluid like proteinase inhibitors, and, postnatally, bioactive components in colostrum and milk, will influence the luminal environment and hence the formation and stability of antigenic molecules. In addition, milk oligosaccharides can bind to viral and bacterial antigens blocking their interaction with host cell-surface glycans, and thus prevent infection or dysbiosis, as well as their translocation into sub-mucosal sites (135). Hence, increased amounts of accessible antigenic molecules in the lumen will add to the enhanced intestinal macromolecular transfer, boosting the exposure of the immune system to these macromolecules during the perinatal period.

#### Forms and Routes of Antigen Transfer

The antigen format, free vs. immune-complex bound, is a factor of importance for the immunological outcome. Thus, transepithelial passage of antigens, bound in complex to maternal IgG and transported via FcRn, has been observed in both rodents and humans, and the subsequent interaction of these immune-complexes with lamina propria antigen presenting cells might be facilitated by their binding to Fc-receptors on these cells (11, 44, 77, 136–138). In fact, microbial antigens, of maternal intestinal origin, in complex with maternal antibodies (antigen-IgG complexes) are transferred via the placenta in primates or via colostrum/milk and intestinal transfer in other species, and hence induce immune activation in the offspring (139–141). Thus, the FcRn receptor appears to be important for the protection of antigens in complex with IgG from degradation and for guiding the antigen through the epithelial cells during transport and sub-epithelial exposure.

Free dietary antigens may also pass materno-fetal barriers, since proteins experimentally fed to pregnant rats have been detected in antigenic form in both amniotic fluid and fetal blood (142). This may affect the immune response in neonates and shift it causing immune activation and inflammation as usually seen in the adult (143, 144) instead of induction of tolerance.

Experiments in neonatal rats have shown that anti-nutritional factors, such as lectins, block intestinal absorption (145), and oral provocation with the lectin PHA severely decreases endocytosis immediately after exposure (146, 147), due to the binding to the epithelial surface (146, 148, 149). Hence, PHA is thought to have blocked epithelial “receptors” needed for endocytosis. In contrast, provocation feeding with a protease caused an instant decrease in receptor-mediated endocytosis, a gradual decrease in the non-specific endocytic pathway and a temporary increase in paracellular leakiness (147).

Changes in paracellular permeability are caused by the disassembling of intestinal tight junctions, regulated by proteins such as claudins, zonulin, and occludin, causing a loosened pore diameter which in turn allows “larger” molecules to pass through (150–154). Tight junction proteins, specifically zonulin, have been associated with activation of the protease-activated receptors (PAR-2) in the intestinal epithelium, which has been linked to the development of cancer and autoimmune diseases (152, 155). Activation of the PAR-2 receptor in the small intestine has also been described as a trigger for pro-inflammatory responses such as immune cell recruitment, mucus secretion, and signaling to enterocytes, immune cells and the enteric nervous system (156–159).

In general, it would seem that the pathway of antigen transfer across the intestinal epithelial barrier could affect the immune system response. Transcellular passage of antigen complexes act as an educational pathway for the newborn's developing immune system, whereas paracellular passage would cause a primary immune response against those free antigens. Hence, during this critical period of early life, there is a continuous balance between tolerance induction and effector immune responses (13).

### Effects of Antigen Transfer on the Immune System

The communication between the luminal content, including microorganisms and dietary macromolecules, and the immune system has been studied mostly with regards to their interaction via the intestinal epithelial cells and their receptors, while less emphasis has been placed on the direct interaction of antigenic molecules after passage across the intestinal epithelium. Moreover, the route for transepithelial passage of antigens, either by the transcellular endocytic pathway (160) or by paracellular “leakage” between the epithelial cells, might also be of importance for the resulting immune response (13). After epithelial passage of luminal antigens and interaction with the local immune system this might either result in tolerance induction or priming of a protective immune response, but could also result in immune activation and inflammation.

Early studies on tissue transplantation suggest that the fetal/neonatal immune system is immature and predisposed to induction of tolerance (161). Transfer of macromolecules or intact antigens across the intestinal epithelium induces differentiation of regulatory T-cells (Tregs) and tolerance, and further protection from allergic diseases. Recently, Ohsaki et al., showed that maternal cutaneous sensitization to an antigen could prevent against antigen-specific IgE production, intestinal mast cell expansion and food allergy in the offspring (162). The observed effects were mediated by neonatal FcRn–mediated transfer of maternal IgG or antigen-IgG immune complexes via breast milk. FcRn-dependent antigen presentation to CD11c+ dendritic cells was also required to induce oral tolerance in the offspring (163).

Dietary macromolecules that induce Treg cells at weaning appear to be required to suppress the immune response to dietary antigens that are important in controlling inflammatory or allergic responses (144). Moreover, maternal IgG against maternal gut microbiota, that are transferred after birth in mice and possible via the placenta in humans, limits the activation of both mucosal T-cells and B-cells against the gut microbiota in her young offspring (164).

Natural weaning has been classified as “physiological inflammation,” with the recruitment of immune cells to the gut, mediated by chemokines and cytokines released by the enterocytes (165). T-cell recruitment to the neonatal mucosa has proven crucial for gut maturation and immune system activation, as well as for the establishment of oral tolerance in early life (80, 166–171). The prominent changes in microbiota induced by weaning seem to be required for immune maturation and the induction of Treg cells, while disturbances in the weaning reaction lead to pathological conditions, with increased susceptibility to allergy and inflammation later in life (172).

As discussed above the route of transepithelial passage of antigens is important in determining the immune response/outcome, where transcellular passage with enterocyte processing and FcRn biding may be preferable compared to paracellular passage (leakage), which instead might result in inflammation and/or sensitization. The high endocytic activity of fetal-type enterocytes and increased macromolecular intestinal permeability might contribute to the increased susceptibility of preterm and term neonates to enteric infection and allergies. The immaturity of the intestine, together with the high epithelial endocytic activity, is possibly involved in the high risk of developing necrotizing enterocolitis (NEC) in preterm infants (173, 174).

## Perspectives and Conclusions

Selecting an appropriate animal species to serve as an ideal model to study the gut and immune system of human infants is not an easy task. The neonatal suckling rat (28, 82) and the newborn pig (175, 176) have often been used for this purpose. The rat is an altricial species, born very immature and with a permeable intestine during the entire suckling period, which is quite different from human infants. The suckling rat should probably rather be used as a model for premature infants. The newborn piglet on the other hand, is born agammaglobulinemic with a highly permeable intestine during the first day of life, which is essential in the acquisition of passive immunity. However, this also differs from that which occurs in newborn human infants. Instead, newborn guinea pigs (21) or rabbit pups, which are precocious species, born with their mothers' immunity and a relative mature gut, exhibiting low intestinal permeability, might be more suitable models for newborn humans. However, the latter species are often considered as less favorable from an experimental, practical and economic perspective, since among other things, the rat and the pig are multiparous species that normally give birth to between 10 and 20 or more offspring. Nevertheless, it is of utmost importance to keep in mind the particularities of each species, and to adapt each model species to the human model, such as the relevance of using piglets before or after ingestion of colostrum, as well as the gestational time in the case of using premature animal models.

This review contributes to a better understanding of how macromolecules of dietary and microbial origin in the intestinal lumen are delivered to the underlying immune system and how this transfer changes during development in perinatal life. Any disturbances in the timing and/or balance of these parallel processes, i.e., intestinal maturation, gut immune maturation and luminal microbial colonization, e.g., due to preterm birth, perinatal infection, antibiotic use or nutrient changes during the neonatal period, might affect the establishment of the immune system in the infant. Understanding the development of intestinal macromolecular permeability, in different animal models, will increase our understanding of intestinal barrier function and provide strategies for the prevention of infection and inappropriate antigen transport affecting the immune response. This might also contribute to a better understanding of the etiology of inflammatory diseases of the gastrointestinal tract and in potential preventive and therapeutic strategies.

## Author Contributions

BW planned the manuscript. BW, EA, and F-JP-C researched and wrote the manuscript and made the figures and tables. BW, EA, KP, SP, and F-JP-C edited the manuscript.

## Conflict of Interest

The authors declare that the research was conducted in the absence of any commercial or financial relationships that could be construed as a potential conflict of interest.
